# Stakeholders’ perspectives of mobile x-ray services in support of healthcare-in-place in residential aged care facilities: a qualitative study

**DOI:** 10.1186/s12877-022-03162-9

**Published:** 2022-08-23

**Authors:** Joanne Dollard, Jane Edwards, Lalit Yadav, Virginie Gaget, David Tivey, Maria Inacio, Guy Maddern, Renuka Visvanathan

**Affiliations:** 1 Adelaide Geriatrics Training and Research with Aged Care (GTRAC) Centre, Adelaide Medical School, Faculty of Health and Medical Sciences, University of Adelaide, Woodville South, SA Australia; 2 Basil Hetzel Institute for Translational Health Research, Central Adelaide Local Health Network, Woodville South, SA Australia; 3 Surgical Specialties, University of Adelaide, The Queen Elizabeth Hospital, Woodville, SA Australia; 4 Royal Australasian College of Surgeons, Adelaide, SA Australia; 5 Registry of Senior Australians, South Australian Health and Medical Research Institute, Adelaide, SA Australia; 6 UniSA Allied Health and Human Movement, University of South Australia, Adelaide, SA Australia; 7 Aged and Extended Care Services, The Queen Elizabeth Hospital, Central Adelaide Local Health Network, Adelaide, Australia

**Keywords:** Nursing homes, Radiography, Delivery of health care, Health care costs

## Abstract

**Background:**

There is interest in reducing avoidable emergency department presentations from residential aged care facilities (RACF). Mobile x-ray services may enable the delivery of healthcare in residential aged care facilities. Accordingly, the Australian Government in November 2019 introduced a Medicare Benefit Schedule rebate providing for a ‘call-out’ fee payable to radiology service providers.

This study aims to understand stakeholder perspectives on the benefits of mobile x-ray services and the factors influencing their adoption by RACFs.

**Design, setting, participants:**

Twenty-two semi-structured interviews were conducted between October 2020 and February 2021 with a range of stakeholders involved in healthcare delivery to residents: a) general practitioners; b) emergency department clinicians; c) paramedic clinicians; d) a hospital avoidance clinician; e) radiology clinicians and managers; and f) aged care clinicians and managers. Thematic analysis was conducted.

**Results:**

Mobile x-ray services were considered valuable for RACF residents. Lack of timely general practitioner in-person assessment and referral, as well as staffing deficits in residential aged care facilities, reduces optimal use of mobile x-ray services and results in potentially unnecessary hospital transfers.

**Conclusions:**

The use of mobile x-ray services, as a hospital avoidance strategy, depends on the capacity of RACFs to provide more complex healthcare-in-place. However, this requires greater access to general practitioners for in-person assessment and referral, adequate staffing numbers and appropriately skilled nursing staff within residential aged care facilities.

## Background

Australia has a well-established residential aged care system, currently spending $14.1 billion per annum on residential aged care services through 830 approved providers [1]. Despite a policy shift towards care at home, the number of people residing permanently in residential aged care has grown, with 243,117 people receiving care as permanent residents in 2020–21; an increase of 1246 from the previous financial year [1]. Residential aged care is provided by for profit agencies, not for-profit agencies and government run facilities. Most recently, the greatest growth in the sector has been among for-profit operators. The Australian government provides subsidies for the care of individual residents, irrespective of the type of operator, and residents pay a small part of the cost of their care [2].

Aged care facilities provide care for activities such bathing, dressing, grooming, toileting and some clinical care, such as wound care and medication administration [2]. The bulk of the aged care workforce are personal care workers (PCWs), who are untrained or have basic training in care work. The workforce also comprises enrolled nurses (who have completed an accredited program of study in nursing and work under the supervision of registered nurses) and registered nurses (who have completed Bachelor level training at an accredited university) [2]. Currently, while each facility has a manager, there are no national standards on mandatory staffing numbers or staff mix within residential aged care facilities (including both nursing staff and PCWs) [3].

The Australian health care system is complex and fragmented [4]. However, it is acknowledged by the Organization for Economic Cooperation and Development to be functioning well and is a hybrid model [5]. It has a universal insurance scheme (Medicare) that provides free or subsidized medical, allied health and psychology care and diagnostic tests [4]. It funds public and subsidizes private hospitals [5]. People may also purchase private insurance and can access private hospital care and private diagnostic services where they may have to pay a gap payment [5]. Public hospitals provide care at no direct cost to individuals and provide the majority of emergency department services [6]. Ambulance services are run by state governments and charge for their services, though people on welfare benefits are not charged, nor are people who pay a small, annual membership fee [7].

General practitioners (GPs), on the other hand, are usually self-employed and provide care on a fee-for-service model (which attracts a Medicare rebate) [4]. GPs are usually not part of the residential aged care services but provide medical services to individual residents on a fee-for-service basis and usually on request from nursing staff within facilities [2]. While many GPs usually regularly visit residents under their care, this is discretionary. GPs are not mandated to visit at specified time periods. GPs also act as gatekeepers to specialist medical care and to many diagnostic procedures, including x-rays [4].

Residents from residential aged care facilities (RACFs) are major consumers of emergency department (ED) services. In the 2018–19 financial year [8], 36.9% of residents of RACF presented to ED and 31.1% of residents were admitted [8]. Falls and associated injuries are the most common reasons for hospitalisation and ED presentations, as are respiratory diseases and diseases of the circulatory system. Demands for ED services are likely to increase among residents unless new models of care that better support the delivery of healthcare-in-place are implemented [9, 10].

Up to 60% of admissions from RACF are avoidable and could be managed in RACFs with the right systems and resources [9]. Remaining in-place for treatment has the potential to reduce individual’s risk of exposure to hospital acquired complications, such as delirium and falls [11].

Hospitalisation, due to falls and infections, have recently been investigated using a multi-disciplinary clinical panel to discuss the root cause of the problem. The studies found that in hospitalisations related to 47 consecutive falls and 49 consecutive infections (59.2% respiratory), timely access to mobile x-ray services (MXS) might have aided in-house diagnosis and reduced hospital transfer [12, 13]. Globally, advances in technology, notably portability and remote monitoring, are making this sort of change in healthcare delivery possible [14, 15]. In Australia, private and state supported MXS have existed for many years. For example, a 2015 evaluation of a MXS attending RACF in one Australian state (Victoria) reported an 11.5% reduction in ED presentations requiring chest, hip and pelvis, spine and abdomen x-rays [16]. This study [16] contributed somewhat to the Australian Government’s decision to subsidize MXS call out fees. In November 2019, recognising the potential of mobile x-rays, the Australian Government introduced a Medicare Benefit Schedule (MBS) rebate for mobile x-ray call-out fee ($73.65) [17], in addition to the usual rebate for x-ray services (e.g. chest (lung fields) x-ray (with the 85% rebate, cost is $41.10). The rebate is available when a resident has experienced a fall or has suspected pneumonia, heart failure, or acute abdomen or bowel obstruction [17] and is assessed in-person by a GP. Only one call-out rebate is payable even if more than one resident of the facility receives MXS during a provider’s visit.

Until now, the applicability of MXS under the current recommendations and funding has not been investigated from the perspective of a range of stakeholders (i.e. clinician and managerial staff from RACFs, radiology services, hospital and ambulance services, as well as general practitioners) involved in the provision of healthcare to RACF residents. This knowledge is a necessary step towards the evolution of an effective service model that can be widely adopted. The present study therefore aimed to explore stakeholders’ perspectives about the value of MXS and identify challenges influencing its adoption.

## Methods

A qualitative and exploratory study was conducted. This design allowed capture of the perspectives of key stakeholders in the absence of relevant and previously published data.

Ethics approval was obtained from the University of Adelaide Human Research Ethics Committee H-2020-197.

### Recruitment

The sampling frame was developed based on the stakeholder groups involved in the care of residents living in RACF. Using our research and clinical networks, we identified businesses (e.g. aged care and radiology providers) and services (e.g. emergency departments and ambulance staff) that were willing to nominate individuals with valuable clinical and management information to be invited to participate in the study (Table 1).

**Table 1:** Category of stakeholders and number of participants (*N* = 22)

Category of stakeholder	N
RACF managers and senior clinical staff	7
General practitioners	3
Hospital avoidance program (Registered Nurse)	1
Senior paramedics and extended care paramedics	3
Emergency Department consultant physician	1
Emergency Department Nurse Unit Manager	1
Emergency Department Clinical Nurse	1
Radiographers (private and public)	3
Radiologist	1
MXR service manager	1

*RACF* residential aged care facility, *MXR* Mobile X-Ray

All stakeholders were emailed an invitation with an attached information sheet and consent form. Following this, a researcher contacted them and, if they consented, a time and date were arranged for a face-to-face or telephone interview. Honorariums were offered to 15 participants ($100 for non-medical staff employed by state government health service; $150 for other stakeholders). The participating RACFs received a $500 honorarium to compensate for releasing staff from normal duties and recruiting participants for a parallel study (which will be reported in subsequent publications).

### Data collection

The interviews ranged in length from 26 to 64 minutes (median 38 minutes) and were held between 27/10/2020 and 16/02/2021. They were guided by a de novo interview schedule (Supporting Information), which was piloted with a stakeholder. All interviews were audio-recorded and transcribed verbatim. The transcripts were then checked against the audio-recordings.

### Analysis

Data were thematically analysed following the six steps advocated by Braun and Clarke [18]. These included reading and re-reading transcripts to become familiar with the data, generating initial codes, deciding on themes, and ensuring that the themes adequately reflected data. Themes were then defined and their contribution to understanding of the data elucidated. A selection of transcripts was independently analysed by three researchers where themes and associated codes were discussed and refined. The remaining transcripts were then coded by one researcher.

Deductive analysis involved analysing transcripts for data that provided information aligned with the research objectives while inductive analysis allowed identification of issues not strictly aligned with the research objectives. This yielded unforeseen and important insight into the research.

## Results

Twenty-seven stakeholders were invited to participate. One declined, one did not respond to the invitation, one withdrew due to illness, and two were unable to participate due to time constraints, leaving 22 participants (see Table 1).

Two themes, resulting from the analysis, are discussed: a) MXS and their dependence on healthcare-in-place and b) factors hindering optimal use of MXS.

### Theme a) MXS and healthcare-in-place

Residential aged care staff and general practitioners (GPs) reflected on the increasingly complex health needs of residents. They considered that RACFs needed to redefine their service to provide more onsite healthcare:

*…aged care is becoming more acute and complex. People aren’t coming in the door low care; they are coming in the door with complex needs and health. I think we need to…start meeting what services we can provide. That can be mobile services for the residents. (1: Residential Services Manager, RACF a)*

Rather than automatically sending people to ED after a fall when they show signs of pain:

*[RACF are] getting GPs involved…and other external services…to get into the home, rather than having to send people to hospital…ECGs [electro cardiographs] and getting added nursing to help with wound care or things like that. (15: Care Manager, RACF d).*

A GP commented on benefits for the health system of expanding the role of RACFs.

*[RACFs have] …become a step down from hospital settings to rehab to palliative care settings…and it would make sense to incorporate it into reducing the burden on the acute care setting…more services would be able to be provided within that facility. (11: General Practitioner)*

Unanimously, MXS was reported to benefit residents by avoiding hospital transfer. Both RACF staff and GPs prefer in-house management because of the potentially adverse consequences for residents going to ED such as worsening symptoms of dementia, delirium, dehydration, pressure sores and injury.

*One poor lady…while she was waiting for an ambulance to bring her back [from ED to RACF] she somehow ‘miraculously’, ‘spontaneously’ fractured her hip (8: General Practitioner).*

Participants working in aged care perceived that MXS could improve the clinical management of residents with a range of conditions: suspected fractures not requiring surgical reduction; abdominal pain and bowel obstruction; and suspected respiratory tract infection. They considered that the MXS could help identify problems, clarifying the necessity of transfer to the ED and expediting appropriate care.

*Do they need to go to the ED or do they need to wait here? (3: Clinical Nurse RACF a)*

*[MXS would be useful]…just to make sure the lungs are clear…get them on some medication if we need to. Then within …24-28 hours, the medication’s kicked in and you have saved a transfer. (3 RACF Manager RACF b).*

A small number of stakeholders raised potential disadvantages for residents associated with MXS (while still endorsing the potential benefits of MXS for residents). Firstly, a small number of families wanted residents transferred to acute care hospitals, rather than being treated in RACF, because of a belief “…that’s where they’re going to be seen properly” (1 Residential care manager RACF a). Another stakeholder considered the possibility that resident’s conditions may change and that adequate symptomatic care may not be provided:

*…the concern is the delay in treatment and exacerbation of the symptoms which warranted the x-ray…Then, obviously, unmanaged pain (13 ED Nurse Unit Manager).*

Another stakeholder wondered about the possibility that poor flow of information regarding the results of x-rays may impede timely care for residents:

*A doctor might remotely be making a request for an x-ray and then waiting for the report…Just someone falling through the cracks and not having the x-ray adequately addressed (16 Ambulance Service Clinician/Manager).*

### Theme b) factors hindering optimal use of MXS

Participants identified two primary challenges to optimal use of MXS. The first was timely access to GP assessment and referral. The second was staffing and skill levels within the RACFs.

The lack of timely access to assessment by GPs was a barrier to the use of MXS. Residential aged care staff reported being able to easily access GPs by telephone, but access to in-person assessment (required for the rebate) was more difficult to organise, especially after hours. Some RACF staff reported that some RACFs do not have enough GPs to respond to residents’ needs in a timely way, and other participants acknowledged GPs’ competing time pressures.

*At the moment, one doctor is covering most of the site because we don’t have enough doctors. (7: Senior Registered Nurse RACF b)*

*…we get told [by GP], I’ve got my clinic, I’ve got this, and I’ve got that. (1: Residential Care Manager RACF a)*

*We can’t contact the doctor most of the time on the weekend. (3: Clinical Nurse RACF a)*

Staff working in RACFs, the ambulance service and ED departments indicated that transfers also occurred when GPs were unable to rely on information relayed via telephone, or when RACF staff regard ED as the way to secure a medical assessment:

*For GPs, who get second-hand assessment from RNs, the ED is a one stop shop for thorough assessment. (16: Ambulance Service Clinician/Manager)*

*…if you cannot get the doctor…you prefer to send the resident to the emergency department. At least you know you’ve got a doctor over there. (3: Clinical Nurse RACF a)*

*… nursing homes will just … send in every patient for an assessment …it’s sometimes very difficult to get a locum…it’s very easy for a locum to turn around, rather than spending maybe 45 minutes with the resident trying to work out what’s going on, it’s just ‘right, send them into ED for assessment’. (13: ED Nurse Unit Manager)*

Staffing and skill levels within the aged care facility hinders optimal use of MXS. Inadequate RACF staffing contributed to the under-utilisation of MXS, when residents’ escalating healthcare needs could not be appropriately managed on-site, resulting in hospital transfer:

*… these aged care staff are just run off their feet on a good day, before anyone takes a topple. You get one palliative patient and one person that’s fallen over, and they [RACF staff] are stretched too thin…. (16: Ambulance Service Clinician/Manager)*

*… their [RACF nursing staff] default becomes 000 because they might be a registered nurse with 120 residents and they’ve got one resident that is unwell and they just don’t have the time or the understanding of how else they can support that patient. (18: Ambulance Service Clinician/Manager)*

Similarly, ED staff reported that sometimes RACF staff were not familiar with residents because of staffing conditions and were more likely to transfer them:

*…it was an agency nurse… it was their first shift in this area or things like that. It’s not an uncommon occurrence where they [RACF nursing staff] might not know what the patient’s baseline is and I know this stems from the inability to attract and retain staff in some aged care facilities. (17: ED Clinical Nurse)*

Some participants (mostly non-RACF staff) also considered that many transfers occurred because RACF nursing staff lack the necessary skills to assess and monitor residents and even to assess whether transfer was necessary.

*If RACF staff were supported to have people assessed and managed in place it would be great. (21: Ambulance Service Clinician/Manager)*

*They need better overall assessment skills. They lose their acute nursing skills…they may have never worked in an acute area…[or]they’ve always worked in age care and they don’t use those acute care assessment skills as much…It’s really simple things that get missed. (22: Hospital Avoidance Clinician)*

*However, in a difficult and busy environment like an RACF, …making sure the patient is monitored accurately is the big concern. (13: ED Nurse Unit Manager)*

## Discussion

This is the first Australian study to explore the perspectives of a wide range of stakeholders on providing MXS in RACFs. However, we note the work of Kjelle et al. who explored the perspectives of managers in the Norwegian context [19]. Participants acknowledged the complex needs of residents, and that MXS could enable residents to be managed in RACFs and potentially avoid hospital transfer. However, timely access to GPs and RACF staffing issues were primary challenges to using MXS because they hindered providing healthcare-in-place. We conclude, based on the insights and experience of stakeholders, that the present healthcare delivery system does not enable RACFs to easily access MXS. This limits the value of MXS in the RACF setting because these problems will not allow the full benefit of MXS to promote “healthcare-in-place”.

The healthcare needs of residents entering RACFs have been increasing over time, and this trend is likely to continue. Residents entering RACF with frailty scores > 0.3 (most frail) have doubled between 2006 and 2015, representing 50% of the new entrants in 2016 [20].

In accordance with the increased complexity of residents’ healthcare needs, there were 1.5 million more GP services between 2005 and 2014 to residents in RACFs [21]. Because most GPs operate independent practices whilst trying to provide service to their patients living in aged care, they may not always be able to provide timely in-person assessments when required. Accordingly, between 2005 and 2014, the proportion of GP consultations in ‘business hours’ decreased by 11.67% (94 to 82%) while after-hours GP consultations increased by 10% (4 to 14%) [21].

The structure of the Medicare call-out fee rebate, which requires in-person GP assessment prior to ordering the MXS, is an impediment to its successful use. If GPs can’t attend RACFs in person, there is an increased likelihood that residents are transferred to hospital for assessment or investigation. MXS are currently also rarely available after hours, resulting in a further barrier to implementation. Allowing GPs to request a MXS following tele-consultation may increase uptake of the Medicare call-out rebate and promote the uptake of timely MXS. Another option is to permit the ordering of MXS by other clinicians, in collaboration with a GP, who could later assess the patient in-person, preferably informed by the results of the MXS. This could be the registered nurses in the RACF, hospital avoidance program staff, nurse practitioners, or extended care paramedics (ECPs). Nurse practitioners located in RACFs promote healthcare-in-place and hospital avoidance [22, 23]. However, in 2016, they constituted a mere 0.3% FTE of RACF staff [3]. Likewise, greater use of ECPs can promote more healthcare-in-place, especially for after-hours care (no data are available about the extent of their use in RACFs) [24].

The workforce numbers and skills within the aged care facility are important. Despite the increased complexity of residents’ healthcare needs, the availability of clinicians in RACFs has paradoxically reduced. Between 2003 and 2016, full-time equivalent numbers have reduced for registered nurses (21 to 14.6%), enrolled nurses (14.4 to 9.3%) and allied health (7.6 to 4%) [3, 21]. Such reductions can make it difficult to manage residents in place, despite efforts to improve overall care management plans and delivery. A strategic intent to enable healthcare to be delivered in RACFs needs to be matched by significant investment in making work in the aged care sector more attractive to skilled clinicians. Substantially greater funding and increased training opportunities are required for clinical staff.

### Limitations

The limitation of this research is that it consisted of a relatively small sample, mostly from one health jurisdiction, limiting the generalisability of the findings. The perspectives of residents and carers on the use of MXS in RACFs have been investigated in other arms of this research project and will be reported in other publications. However, our findings are the first qualitative assessment from a wide range of stakeholders, but further confirmation through qualitative research in other health jurisdictions would be beneficial.

## Conclusion

Our study confirms strong support by stakeholders for MXS in RACFs, for some health scenarios, because they consider resident outcomes are better and detrimental effects of hospitalisation can be avoided. Our participants consider that for MXS to be widely adopted, RACFs must be able to provide healthcare-in-place. They identified barriers to this development. One is timely access to in-person GP assessment and referral. The second is adequate staffing and appropriately skilled nurses in RACFs. These need to be addressed from a systems perspective. Addressing a single element [25], such as expanding rebates for MXS, without attention to other deficiencies, such as skilled staff in sufficient numbers, is unlikely to reduce potentially avoidable hospital transfers.

## Data Availability

Requests for data should be directed to the corresponding author (renuka.visvanathan@adelaide.edu.au) and ensuing research will require collaboration with the chief investigators (RV and JD). Any requests will be assessed for scientific rigour by this investigator team. A request for ethics approval and/or amendment must be prepared by the requestor in line with the requirements of Human Research Ethics Committee of the University of Adelaide. This ethics approval/amendment must be approved by the Human Research Ethics Committee of the University of Adelaide. A data sharing agreement will need to be put in place. The requestor will be responsible for providing the necessary funding required for this process, including for the provision of data. Given that the grant funding for the project is in place to the 31st of December 2022 and there may be analyses continuing, then data sharing is embargoed till the 30th of March 2023.
